# Impact of Dose, Sex, and Strain on Oxaliplatin-Induced Peripheral Neuropathy in Mice

**DOI:** 10.3389/fpain.2021.683168

**Published:** 2021-07-22

**Authors:** Urszula O. Warncke, Wisam Toma, Julie A. Meade, Abigail J. Park, Danielle C. Thompson, Martial Caillaud, John W. Bigbee, Camron D. Bryant, M. Imad Damaj

**Affiliations:** ^1^Department of Pharmacology and Toxicology, Medical College of Virginia Campus, Virginia Commonwealth University, Richmond, VA, United States; ^2^Wright Center for Clinical and Translational Research, Virginia Commonwealth University, Richmond, VA, United States; ^3^Department of Anatomy and Neurobiology, School of Medicine, Virginia Commonwealth University, Richmond, VA, United States; ^4^Laboratory of Addiction Genetics, Department of Pharmacology and Experimental Therapeutics and Psychiatry, Boston University School of Medicine, Boston, MA, United States

**Keywords:** oxaliplatin, neuropathy, strain differences, pre-clinical CIPN model, behavioral assay

## Abstract

Chemotherapy-induced peripheral neuropathy (CIPN) is a common, dose limiting, and long-lasting side effect of chemotherapy treatment. Unfortunately, no treatment has proven efficacious for this side effect. Rodent models play a crucial role in the discovery of new mechanisms underlying the initiation, progression, and recovery of CIPN and the potential discovery of new therapeutics. However, there is limited consistency in the dose, the sex, age, and genetic background of the animal used in these studies and the outcome measures used in evaluation of CIPN rely primarily on noxious and reflexive measures. The main objective of this study was to provide a comprehensive and systematic characterization of oxaliplatin-induced peripheral neuropathy in mice by using a battery of behavioral, sensory, electrophysiological, and morphometric measures in both sexes of the two widely used strains of mice, C57BL/6J and BALB/cJ. Mice received intraperitoneal injections of 3 or 30 mg/kg cumulative doses of oxaliplatin over the course of 2 weeks. Both doses induced long-term and time-dependent mechanical and cold hypersensitivity. Our results show that 30 mg/kg oxaliplatin reduced the locomotor activity in C57BL/6J mice, and C57BL/6J females showed anxiety-like behavior one-week post completion of treatment. In the same dose group, BALB/cJ males and females sustained a larger decrease in sucrose preference than either male or female C57BL/6J mice. Both strains failed to show significant changes in burrowing and nesting behaviors. Two clinically relevant assessments of changes to the peripheral nerve fibers, nerve conduction and intraepidermal nerve fiber density (IENFD) were evaluated. Only BALB/cJ females showed significant reduction in the nerve conduction amplitude 1 week after 30 mg/kg oxaliplatin regimen. Moreover, this dose of the chemo agent reduced the IENF density in both sexes and strains. Our findings suggest that mouse strain, sex, and assay type should be carefully considered when assessing the effects of oxaliplatin and potential therapeutic interventions.

## Introduction

Oxaliplatin is a platinum-based chemotherapeutic agent used against digestive tract tumors, esophageal, liver, pancreatic, and colorectal cancers (CRC) (1–4). Despite its high efficacy at fighting cancer, this drug is considered one of the most neurotoxic chemotherapeutics, where ~90% of patients develop acute neuropathy symptoms which appear hours after infusion (5). The feature characteristics of oxaliplatin-induced peripheral neuropathy (OIPN) are cold hypersensitivity, allodynia, pain, sensory ataxia, and dysesthesia in the distal extremities and perioral regions (6). The negative impact of OIPN on continuation of treatment can be substantial leading to treatment delay, dose reduction, or cessation (7). In a small clinical study, 40% of colorectal cancer patients treated with oxaliplatin aborted their treatment due to neurotoxicity (8). Moreover, due to oxaliplatin's cumulative toxicity, up to half of patients treated with this chemotherapeutic progress to chronic peripheral neuropathy, which can persist for years after discontinuation of the therapy (9–12).

With the expanding medical use of oxaliplatin, an increasing number of patients are at risk of developing OIPN and other signs of neurotoxicity that burden their quality of life. Such signs include fatigue, distress, anxiety, and depression. Emotional distress reported in colorectal cancer patients treated with 6 cycles of oxaliplatin resulted from inability to cope with neuropathic symptoms and impairment of performing activities of daily living (13). Additionally, Bonhof et al. (14) reported that CRC survivors with high chemotherapy-induced peripheral neuropathy scores experienced more anxiety, depressive symptoms, and fatigue than survivors with lesser neuropathic scores. Overall, the prevalence of depression and anxiety in cancer patients is 58 and 11.5%, respectively (15, 16). Other affective disturbances associated with pain involve reduced appetite, anhedonia, disruptions to sleep cycles, and impaired social interactions (17).

To this day, there are no effective preventative measures or treatments against OIPN. The population of patients with neuropathies is rising and the search for new therapeutics that could significantly improve the quality of life and tolerance of cancer medications is continuing. In that regard, the use of animal models allows for studying variables influencing the progression of neuropathy and recovery, and evaluation of new preventive and therapeutic approaches for OIPN. However, studying OIPN in animal models has its own challenges. The majority of chemotherapy-induced peripheral neuropathy studies in rodents are heterogeneous in nature with respect to strain, sex, dosage, route of administration, duration of treatment, and outcome measures which hinder comparison between studies (18). Genetic diversity among different strains of rodents can influence responses to pain and neuropathy assays (19). Additionally, the use of a single strain of inbred mice might not provide sufficient evidence for drug efficacy in clinical settings. As such, a recent study that looked at the susceptibility of different mouse strains (BALB-c, C57BL/6, DBA/2J, A/J, FVB and CD1 from Envigo) to OIPN-associated phenotypes found that morphometric, electrophysiological, and cold hypersensitivity measures are strain-dependent (20).

Assessing complex OIPN pain phenotypes in rodents is challenging due to is multimodality in the sensory, behavioral, and cognitive aspects in humans (21, 22). In that regard, relying upon stimulus-evoked assessment of pain sensitivity in animals might be insufficient for moving preclinical findings into clinical settings. In order to study intensity and relief of chemo-induced neuropathy, it is important to test additional endpoints which are relevant to human conditions and experiences. We recently reported that paclitaxel treatment in mice induced alterations in some affective-related behaviors which include nest building, burrowing, wheel running, anxiety- (light/dark box test), depression-, and anhedonia- (sucrose preference test) like behaviors (23).

The primary objective of this study was to provide a more comprehensive and systematic characterization of oxaliplatin-induced peripheral and painful neuropathy in mice. To accomplish this goal, both sexes of the two widely used strains of mice, C57BL/6J and BALB/cJ (24), were subjected to intraperitoneal injections of two doses of oxaliplatin. While most studies focus on measuring evoked sensory changes, we investigated the impact of the antineoplastic agent on several behavioral measures. Moreover, we evaluated two clinically relevant neuropathy markers, nerve conduction and intraepidermal nerve fiber density, in C57BL/6J and BALB/cJ male and female mice. This study aims to bridge the gaps in the literature between different experimental parameters in chemotherapy treatment, strain, sex, and dose, using a battery of assays in two inbred mouse strains frequently used in cancer research.

## Methods

### Animals

Experiments were performed on adult male and female C57BL/6J and BALB/cJ mice. All mice were supplied from The Jackson Laboratory (Bar Harbor, ME). Mice were 12 weeks old at the beginning of the experiments. C57BL/6J mice were group-housed, with the exception of a separate cohort of mice which was single-housed to perform nesting and sucrose preference studies. BALB/cJ male mice were housed one or two per cage due to excessive fighting. Animals were housed in a climate-controlled room on a 12 h light/dark cycle (lights on 07:00 h) with *ad libitum* access to chow (Envigo, Teklad LM-485 mouse/rat sterilizable diet, cat # 7012, WI, USA) and water. Cages contained compressed cotton nestlets and cardboard tunnels for enrichment items. All studies were approved by the Institutional Animal Care and Use Committee of Virginia Commonwealth University and followed the National Institutes of Health Guidelines for the Care and Use of Laboratory Animals.

Mice were handled for 1 week and habituated to the room prior to testing. Baseline nociception and affect-like behavior measurements were established. Experiments were performed during the light cycle by blinded female experimenters. To increase scientific rigor, behavioral and electrophysiological changes we measured in several cohorts of mice to reduce the stress associated with frequent testing and handling.

### Drugs and Induction of OIPN

Pharmaceutical grade oxaliplatin (5 mg/ml, Accord, NDC 16729-332-05) was prepared daily in a vehicle of sterile 5% dextrose solution (Hospira, Lake Forest, IL). Animals received intraperitoneal (i.p.) injections of vehicle, low-dose (0.3 mg/kg) or high-dose oxaliplatin (3 mg/kg) for 5 consecutive days, followed by 5 days of rest, followed by a second cycle of five daily injections, as per the method of Ta et al. (11). The total cumulative doses of oxaliplatin over the course of 10 injections was 3 mg/kg for the low-dose regimen and 30 mg/kg for the high-dose regimen. The low and high dosage of the drug were selected based on the literature evidence which indicate their effectiveness in inducing neuropathic pain without unspecific systemic toxicity (11). Moreover, the high dose used in this study is relevant to clinical dosing. Human equivalent dose conversion factors were applied to show dosage relevance for oxaliplatin. The highest recommended dose of oxaliplatin patients is 110 mg/m^2^ (2.97 mg/kg) (25). The total human equivalent dose (THED) of oxaliplatin daily dosing ranges in mice 0.04–10.0 mg/kg/day, and the cumulative 3.0–30.0 mg/kg (0.24–2.4 mg/kg THED) (26–28). Our calculation shows that 30 mg/kg of oxaliplatin is equivalent to 1,110 mg/m^2^ in humans. Both strains and sexes received vehicle, low-dose oxaliplatin, and high-dose oxaliplatin.

### General Experimental Design

**Figure 1:**
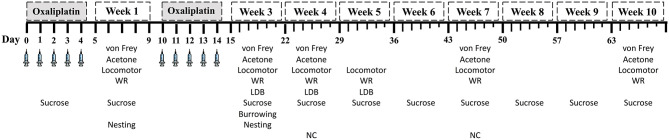
Experimental testing schedule. Weeks 1 and 2 spanned 5-day intervals. Starting at week 3, testing week span for 7 days. LDB, light/dark boxes; WR, wheel running; NC: caudal nerve conduction.

The first wave of five injections were considered week 0. The 5-day rest period between the first and second wave of treatment was designated as the 1st week of testing (week 1). The 3rd week of testing began 24 h following the 10th injection. Week 3 and subsequent testing intervals span for 7 days. If two tests were performed on the same day, the least stressful test occurred first to minimize the influence of one test on the successive test. No more than two tests were performed per day. A larger number of animals was assigned to the high-dose groups to account for potential severe adverse effects from the treatment. Therefore, the number of mice tested was variable across treatment groups and time points. The experimental timeline of behavioral tests, nerve conduction, and humane endpoints are summarized in Figure 1.

### Assessments of Stimulus-Evoked Nociceptive Behaviors

#### Mechanical Hypersensitivity (von Frey Test)

Mechanical hypersensitivity thresholds were determined using manual von Frey filaments according to the methods of Chaplan et al. (29). Shortly, each animal was placed in a Plexiglas chamber on an elevated mesh and acclimated for at least 30 min. Mechanical force was applied in increasing amounts to the ventral surface of the hind paw until the subject demonstrated a paw withdrawal response whereby the force is expressed in grams. The following number of mice were used in the von Frey test: C57BL/6J, males *n* = 17 and females *n* = 11; male BALB/cJ vehicle and low-dose *n* = 7, high dose n = 9; female BALB/cJ *n* = 10 females per group. All mice were tested at baseline, and weeks 1, 3, 4, 7, and 10 of the study.

#### Cold Hypersensitivity (Acetone Test)

Similar to the von Frey test, the acetone test was performed by habituating C57BL/6J mice in Plexiglas chambers on mesh metal flooring for at least 30 min. Twenty μL of acetone (Fisher Bioscience) was projected onto the ventral surface of the hind paw from a 200 μL pipette (Eppendorf). Total time lifting, licking, or clutching each paw was recorded over the course of 60 s. C57BL/6J male mice (*n* = 8 per group) and female mice (*n* = 8 per group) were tested on weeks 1, 3, 4, 7, and 10. Cold hypersensitivity was not obtained in BALB/cJ mice due to hypermobility of the animals on the mesh which led to an inability of the tester to perform rigorous testing.

### Assessments of Spontaneous Behaviors

#### Locomotor Activity (Locomotor)

Mice were individually placed into Omnitech (Columbus, OH) photocell activity cages (28 × 16.5 cm). Photocell beams interruptions, which assess walking and rearing, were recorded for 30 min. Data are expressed as the average number of beam breaks. Two groups (Group 1 and Group 2) of C57BL/6J animals were tested at different time points. Group 1 (*n* = 9 males/group, *n* = 6 females/group) was assayed at baseline, and week 1, 3, and 5, while Group 2 (*n* = 8 males/group, n = 8 females/group) testing occurred at baseline and week 4, 7, and 10. BALB/cJ males (*n* = 7–9) and females (*n* = 10) locomotor activity were assessed at baseline and week 1, 3, and 5.

#### Voluntary Wheel Running (WR)

Mice were placed in free-standing running wheels (68 cm circumference) with the ability to spin in either direction. After a 5 min acclimation period to the wheels, the wheels were latched allowing for unidirectional spinning for a 2-h session. Rotations were converted to distance traveled by multiplying the number of rotations by the wheel circumference. Data are expressed as the average distance traveled (m). The mice in this voluntary wheel running study were the same subjects as the locomotor activity cohorts, undergoing the same baseline and weekly testing schedule (on different days of the week than locomotor activity).

#### Light/Dark Box Test

The light/dark box test was used to assess anxiety-like symptoms. This assay is based on a conflict between rodents' natural aversion to brightly-illuminated areas and their spontaneous exploratory activity (30). In brief, the LDB apparatus consisted of a small, enclosed dim “dark” box (36 × 10 × 34 cm) with an opening (6 × 6 cm) leading to a larger, brightly illuminated “light” box (36 × 21 × 34 cm). The mice were acclimated to the testing room for 30 min prior to testing. Mice were placed in the light compartment and allowed to explore the apparatus for 5 min. The total time (s) spent in the light compartment was recorded via a video monitoring system and measured by ANY-MAZE software (Stoelting Co., Wood Dale, IL). To assess spontaneous exploratory behavior at different time points in C57BL/6J mice without habituation to the LDB apparatus, Cohort 1 (*n* = 9 sex/group) was tested at baseline and week 3, while Cohort 2 (*n* = 8 sex/group) was tested at week 4 and 7. BALB/cJ males (*n* = 7–9) and females (*n* = 10) were tested at baseline and week 3 and 5.

#### Two-Bottle Choice Test (Sucrose Preference Test)

The sucrose preference test theoretically assesses anhedonia-like behavior (31). Mice were housed individually with *ad libitum* access to food. Mice were presented with two sipper tubes, one containing normal drinking water and the other containing 3% sucrose solution (w/v, Sucrose ≥ 99.5% (GC) Sigma, United States, cat# S7903). The total volume consumed from each tube was measured after 24 h. The position of both sipper tubes was switched for each drinking session to avoid place preference. Sucrose preference was calculated as a percentage of 3% sucrose volume consumed over the total fluid intake, multiplied by 100%. Preference is reported as the average per group. Measurements were collected daily during the first course of 5 injections, then after 10th injection, followed by weekly testing. Sucrose preference was measured in a separate cohort of vehicle-treated and high-dose oxaliplatin-treated C57BL/6J mice (*n* = 10 group/sex). Due to a severe eye infection and tail injuries, one low-dose and one high-dose oxaliplatin BALB/cJ male mice were euthanized, resulting in sample sizes of *n* = 4–6. Female BALB/cJ mice sample size was 6/group.

#### Nesting

The nesting test assesses the impact of spontaneous nociception on activities of daily living. The assay was adapted as previously described by Oliver et al. (32) with some modifications. Briefly, the test was performed on individually-housed mice. All previous nesting material and enrichment items were removed from the home cage prior to conducting the nesting assay. For each cage, two compressed cotton nestlets were cut in half, for a total of 4 rectangular pieces of equal size placed in each cage corner. The mice were allowed 120 min to nest in a quiet room. Nest quality was scored between 1 (no nestlet consolidation) and 5 (complete nestlet consolidation). The nesting assay was conducted in the same subjects as the sucrose preference test. The test was performed weekly for 5 consecutive weeks. Data are expressed as the average nesting score per group.

#### Burrowing Test

Like the nesting test, the burrowing test is another means of assessing voluntary spontaneous behaviors necessary for survival. The burrowing test was performed as previously described with some modifications (33, 34). Long, gray, PCV tubes (20 cm long x 7 cm in diameter) with an upright tilt of ~10° and sealed bottom ends were filled with ~180 grams of clean corncob bedding. Tubes were placed in rat cages (37 × 26 × 19 cm, L × W × H), with the tube opening facing toward the wall. Rat cages contained clean corncob bedding on the floor but lacked food and water. Mice were placed in the rate cages for 30 min sessions. Data are represented as the average amount of bedding displaced (g). Subjects from the sucrose preference test used in burrowing experiments at baseline and week 3 and 5.

### Assessment of Peripheral Nerves

#### Immunohistochemistry and Quantification of Intra-Epidermal Nerve Fibers

Mouse hind paws were removed and placed in freshly prepared PLP fixative (35) at 4°C for 24 h. The glabrous skin on the ventral surface of the hind paws was excised and submerged in 30% (w/v) sucrose at 4°C overnig. The tissues were embedded in Optimal Cutting Temperature embedding medium for frozen tissues (ThermoFisher Scientific) and sectioned at 25 μm on a cryostat. Sections were immersed in cold acetone (−20°C) for 20 min, washed with PBS, and incubated at room temperature for 45 min in blocking solution (5% normal goat serum and 0.3% Triton X-100 in PBS). Sections were incubated with a 1:200 dilution of the primary rabbit anti-mouse polyclonal PGP9.5 antibody (ProteinTech, cat# 14730-1-AP, IL, USA) overnight at 4°C in a humidified chamber. Following three PBS washes, sections received a second blocking step and then incubated for 2 h at room temperature with a 1:300 dilution of goat anti-rabbit IgG (H+L) secondary antibody conjugated with Alexa Fluor® 594 (Life Technologies, cat# A11037, OR, USA). Sections were mounted with Vectashield (Vector Laboratories, Burlingame, CA, USA) and examined using a Zeiss Axio Imager A1 – Fluorescence microscope (Carl Zeiss, AG, Germany). The IENFs of each paw section were counted under 63 × magnification in a blinded fashion, and the density of fibers was calculated as fibers/mm. The mean of IENF density (n= 6/sex/group) was calculated from six mice.

#### Caudal Nerve Conduction

Nerve conduction velocity (NCV) and sensory nerve action potential amplitudes (SNAP) of the caudal nerve were recorded with PowerLab 4/35 electromyography system (AD Instruments, Inc., CO, USA). This test measures amplitude and latency of the evoked response, which are used to calculate nerve conduction velocity. The protocol was adapted from Marmiroli et al. (20). Anesthesia was induced in a chamber with 4% isoflurane carried in oxygen and maintained with 2.5% isoflurane by nose cone during the nerve conduction procedure. Needle recording electrodes (cathode and anode) were inserted 5 mm distal from the base of the tail, with the stimulating electrodes 5.0 cm apart from the recording points (distance measured from cathode to cathode). There was a ground electrode between the stimulation and recording electrodes. The nerve was stimulated with single square-wave pulses of 0.1 ms duration at 7 mA, with a repeat rate of 1 Hz. The neurophysiological measures were conducted under standard conditions in a temperature-controlled room. NCV was calculated by dividing the distance between the recording and stimulating electrodes (0.05 m) by the latency between the stimulus artifact and the onset of the first peak of the elicited action potential latency of the sensory nerve action potential. NCV (m/s) and SNAP (μV) were measured 1 week after the 10th injection (week 4). C57BL/6J mice (*n* = 8 males/group, *n* = 8 females/group) and one cohort of BALB/cJ mice (*n* = 7–8 males/group, *n* = 10 females/group) were used for these measurements.

### Statistical Analyses

The data analyses were carried out with GraphPad Prism software, version 8 (GraphPad Software, Inc., La Jolla, CA) and the data are expressed as mean ± SEM. Statistical analyses were performed as unpaired *t*-tests to compare behaviors of vehicle- and oxaliplatin-treated mice at a single time point, one-way (treatment) or two-way (time x treatment) analysis of variance (ANOVA) with repeated measures or mix effect analysis. When appropriate, Dunnett's or Tukey's *post-hoc* comparison tests were performed. Nesting test scores were analyzed by employing Mann-Whitney test with correction for multiple comparison using the Hold-Sidak method. A three-way ANOVA with repeated measures was performed to investigate (a) differences between sexes of each mouse strain, and (b) strain differences of same sex between both strain. Time and treatment were common factors for each comparison. *p* < 0.05 was considered statistically significant.

## Results

### Oxaliplatin Has Strain- and Sex-Dependent Effects on Body Weight

**Figure 2:**
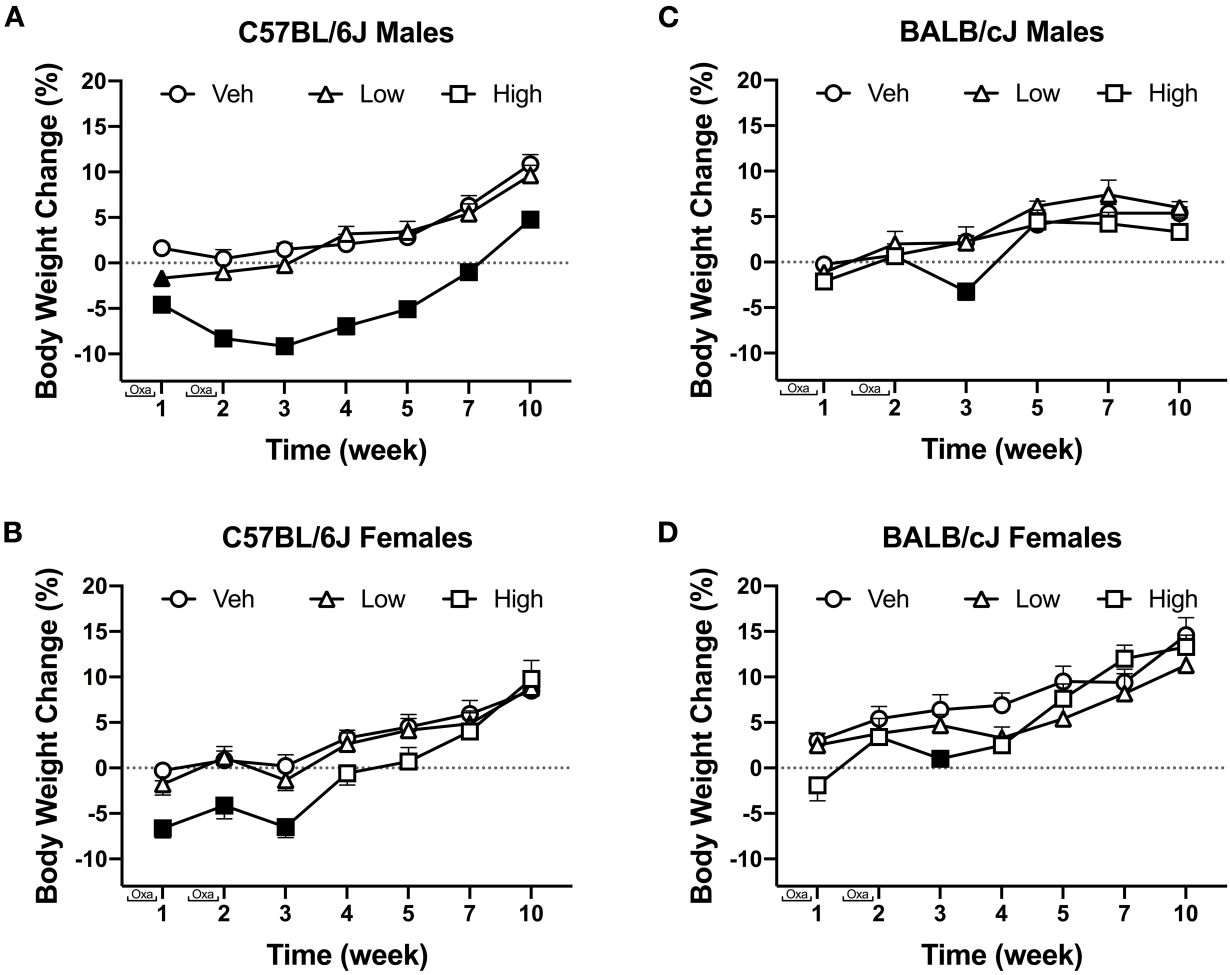
The effect of oxaliplatin on body weight changes from baseline over the course of a 10-week observation period. C57BL/6J males (*n* = 14/group) **(A)** C57BL/6J females (*n* = 14/group) **(B)** BALB/cJ males (*n* = 7–9/group) **(C)** BALB/cJ females (*n* = 10/group) **(D)**. Filled points indicate times at which body weight was statistically different from vehicle. Values are expressed as mean ± SEM. Results were compared using two-way ANOVA (Treatment, Time as RM) for each strain and for each sex followed by Tukey's *post-hoc* test, p < 0.05.

Oxaliplatin induced strain- and sex-dependent effects on weight change. Oxaliplatin had transient effects of body weight in C57BL/6J mice (Figure 2). Vehicle and low-dose treated male C57BL/6J mice steadily gained weight over time. The high-dose oxaliplatin regimen caused a robust weight loss and slowed weight gain, as compared to vehicle controls [*F*_(12, 234)_ = 3.615, *P* < 0.0001, Figure 2A]. Tukey's multiple comparisons test identified significant weight loss on week 1, 2, and 3, with gradual weight gain starting at week 4. The high dose oxaliplatin-treated male C57BL/6J mice returned to baseline weights by week 7 and continued to have stunted weight gain through 10 weeks (*P* < 0.0001) compared to vehicle-treated animals. Likewise, female C57BL/6J mice treated with the high dose of oxaliplatin lost 6% their body weight during the injection periods, which was a statistically significant change [*F*_(12, 234)_ = 2.743, *P* = 0.0017], however, they returned to their baseline measures 1 week after the last injection and caught up with vehicle controls by week 7. The low-dose treated females showed nearly identical profile of body weight gain as the vehicle group (Figure 2B). High-dose oxaliplatin treatment induced a more profound body weight reduction in male C57BL/6J than female C57BL/6J or male BALB/cJ mice [*F*_(1, 52)_ = 6.360, *P* = 0.0148; *F*_(1, 41)_ = 21.39, *P* < 0.0001; respectively; Supplementary Tables 1A,B].

Overall, comparable to C57BL/6J mice, vehicle-treated BALB/cJ mice steadily gained weight over the course of the study. Similarly, low dose oxaliplatin did not affect body weight in male nor female BALB/cJ mice. The high-dose oxaliplatin treatment significantly reduced body weight in male BALB/cJ mice after the second wave of injections (week 3), as compared to vehicle controls [*F*_(10, 105)_ = 2.525, *P* = 0.02, Figure 2C]. This effect on body weight was transient as the BALB/cJ male mice rapidly regained weight and had body weights indistinguishable from vehicle controls by week 5. While the high-dose oxaliplatin regimen did not cause weight loss in female BALB/cJ mice, it stunted their weight gain, as compared to vehicle controls [*F*_(12, 162)_ = 3.438, *P* = 0.0002]. Tukey's multiple comparisons test identified significant effects of high-dose oxaliplatin on preventing weight gain in female BALB/cJ mice at week 3 and week 4, though these oxaliplatin-treated mice caught up to vehicle controls by week 5 (Figure 2D).

### Assessments of Stimulus-Evoked Nociceptive Behaviors

#### Oxaliplatin Induces Long-Term and Dose-Dependent Mechanical Hypersensitivity

**Figure 3:**
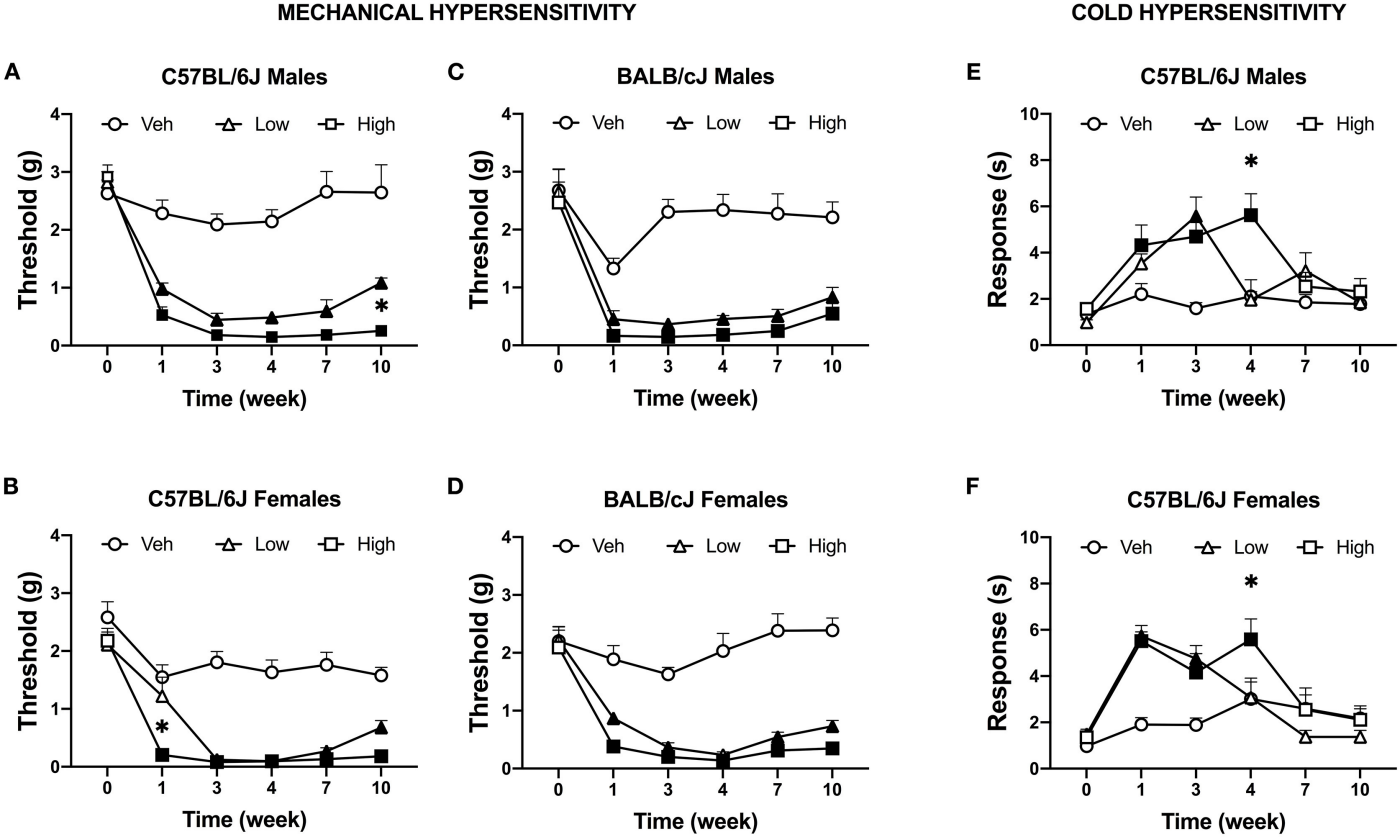
Induction of mechanical and cold hypersensitivity by low and high dose of oxaliplatin. Development of mechanical hypersensitive was monitored after each regimen of oxaliplatin (5 injections of 0.3 or 3 mg/kg) with the von Frey test in C57BL/6J males (*n* = 17/group) **(A)**, C57BL/6J females (*n* = 11/group) **(B)**, BALB/cJ males (*n* = 7–9/group) **(C)**, BALB/cJ females (*n* = 10/group) **(D)**. Similarly, cold hypersensitivity was tested on the same week in in C57BL/6J males (*n* = 8/group) **(E)**, C57BL/6J females (*n* = 8/group) **(F)**. Values are expressed as mean ± SEM. Results were compared using two-way ANOVA (Treatment, Time as RM) for each strain and for each sex followed by Tukey's *post-hoc* test, *p* < 0.05. Filled points indicate times at which mechanical hypersensitivity was statistically different from vehicle. *p < 0.05 high vs. low dose.

We tested the effects of two doses of oxaliplatin on the time-course of stimulus-evoked nociception-related behaviors. No significant difference in mechanical threshold was observed between groups at baseline. Both doses of oxaliplatin caused a comparable long-term mechanical hypersensitivity in both sexes of both strains of mice (Supplementary Tables 1A,B). Five injections of 3.0 mg/kg oxaliplatin reduced mechanical thresholds in all subjects, regardless of sex or strain. Further, all subjects treated with 10 injections of oxaliplatin (low-dose and high-dose) exhibited mechanical hypersensitivity for up to 10 weeks. Male C57BL/6J mice treated with low and high dose of oxaliplatin developed significant mechanical hypersensitivity *F*_(10, 240)_ = 10.52, *P* < 0.0001; Figure 3A), however, the low-dose treated males showed significant difference from the high-dose group at week 11 (*P* = 0.0105). Female C57BL/6J mice developed mechanical hypersensitivity following high-dose oxaliplatin treatment [*F*_(10, 155)_ = 5.896, *P* < 0.0001; Figure 3B]. Interestingly, administration of the low dose regimen didn't decrease the mechanical threshold at week 1 time point. No difference was found between the low- and high-dose treatments at weeks 3 through 10. BALB/cJ males treated with the low- or high-dose oxaliplatin were significantly different from the control vehicle group after the first 5 injections and remained hypersensitive until the end of experiment [*F*_(10, 105)_ = 5.797, *P* < 0.0001; Figure 3C]. Likewise, the mechanical threshold of BALB/cJ females subjected to both the low- and high doses of oxaliplatin reached significantly reduced mechanical threshold at week 1 and remained low until the end of the experiment [*F*_(10, 125)_ = 8.335, *P* < 0.0001; Figure 3D].

#### Oxaliplatin Induces Cold Hypersensitivity in C57BL/6J Mice

Cold hypersensitivity is a hallmark of oxaliplatin-induced neuropathy, and it that can be measured in rodents via acetone test. A dose-dependent effect on the time course of cold hypersensitivity was observed in male C57BL/6J mice [*n* = 8 per group; *F*_(10, 105)_ = 3.852, *P* = 0.0002]. The low-dose of oxaliplatin treatment significantly increased the duration of acetone-elicited behaviors as compared to vehicle control at week 3 only (*P* < 0.0001; Figure 3E). The high-dose group showed an increased in responses to acetone at weeks 1, 3, and 4 (*P* = 0.024, *P* = 0.0005, and *P* < 0.0001, respectively). Corresponding results were found in female C57BL/6J mice (Figure 3F). The low dose significantly increased response time in female C57BL/6J mice after the 1st and 2nd week of injections [*n* = 8 per group; *F*_(10, 105)_ = 4.011, *P* = 0.0001; *P* < 0.0001, *P* = 0.0012], while the high dose of oxaliplatin prolonged the duration of acetone-elicited behaviors until week 4 (*P* = 0.041). Excess locomotion activity of male and female BALB/cJ on the elevated mesh hindered performing the acetone test, hence no data is available.

### Oxaliplatin Has Strain-, Sex-, and Assay-dependent Effects on Spontaneous Behaviors

#### Oxaliplatin Reduces Locomotor Activity in C75BL/6J Male Mice

**Figure 4:**
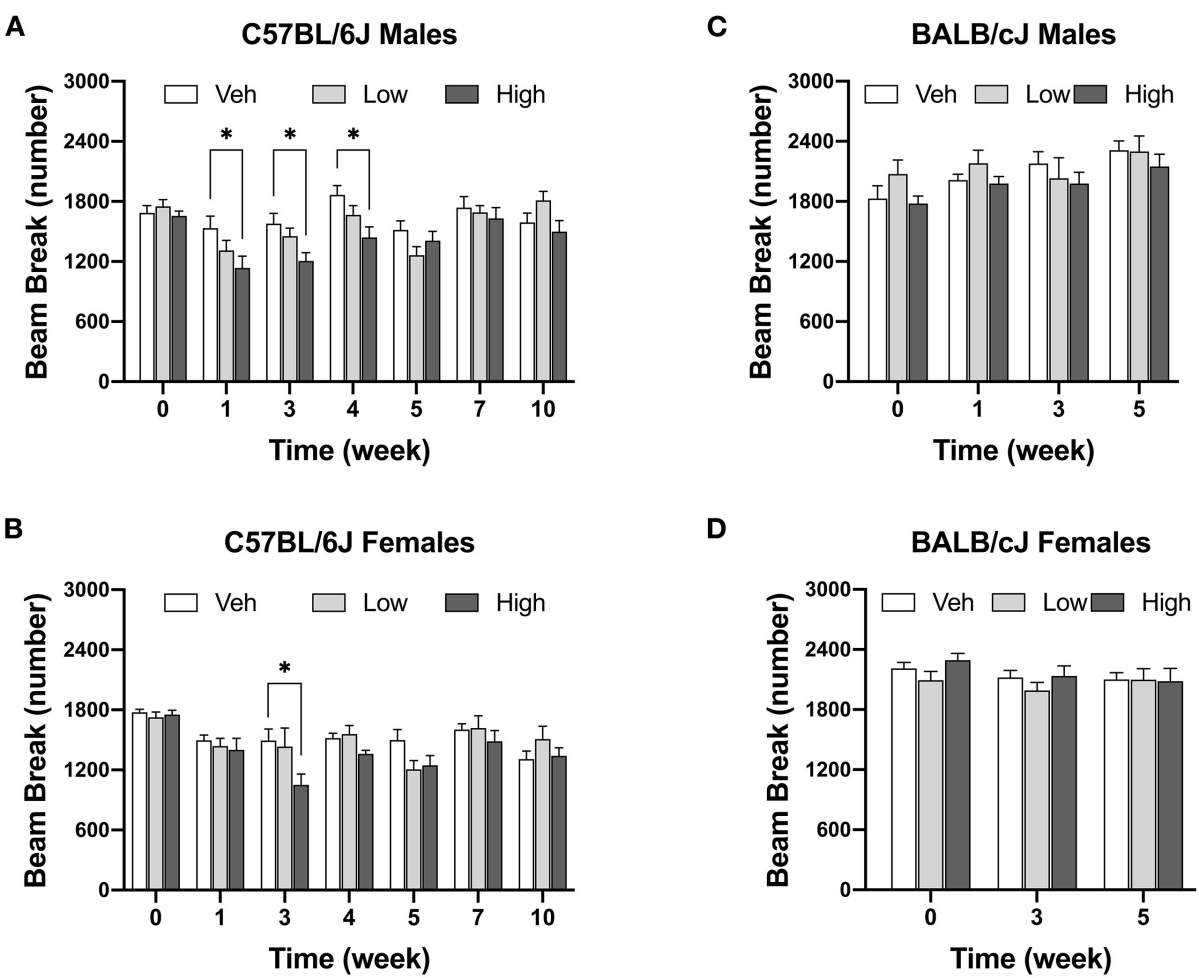
High dose of oxaliplatin reduce spontaneous locomotor activity in C57BL/6J mice. One regimen of the high dose of oxaliplatin was sufficient to reduce locomotor activity in C57BL/6J males (*n* = 8–10/group) **(A)** which continued 1 week after the treatment. C57BL/6J females (*n* = 6–8/group). **(B)** showed lower activity after two regimens. No alteration in motor coordination was obsessed in BALB/cJ males (*n* = 7–9/group) **(C)** nor females (*n* = 10/group) **(D)**. Overall effects of oxaliplatin treatment were identified by two-way or mixed-analysis model ANOVA (Treatment, Time as RM) for each strain and for each sex with *post-hoc* Tukey's multiple comparisons test, *p < 0.05.

The high dose of oxaliplatin significantly reduced locomotor activity in male C57BL/6J mice [*F*_(12, 134)_ = 1.915, *P* = 0.0377]. Decreases in locomotor activity were observed after completion the first and second regimens as compared to vehicle controls. This reduction was still observed 1 week post last injection of the drug (Figure 4A). Although, there was a significant effect of time [*F*_time_ (6, 114) = 13.67, *P* < 0.0001], and dose [*F*_dose_ (2, 45) = 4.75, *P* = 0.0134], with no significant interaction between time and dose [*F*_interaction_ (12, 114) = 1.506, *P* = 0.1218] in female C57BL/6J mice, as revealed by two-way ANOVA analysis. In addition, the Tukey post hoc analysis indicated a decrease in the number of beam breaks in female C57BL/6J mice as compared to vehicle controls at week 4 (*P* = 0.0053, Figure 4B). In contrast, no alterations in the locomotor activity were observed in BALB/cJ males and females after administration of the two doses of oxaliplatin till week 5 of testing. Strain comparison indicated a significant difference between female C57BL/6J and female BALB/cJ mice treated the high dose of oxaliplatin [*F*_(1, 104)_ = 7.881, *P* = 0.006, Supplementary Table 1B].

#### Oxaliplatin Does Not Affect Voluntary Wheel Running

**Figure 5:**
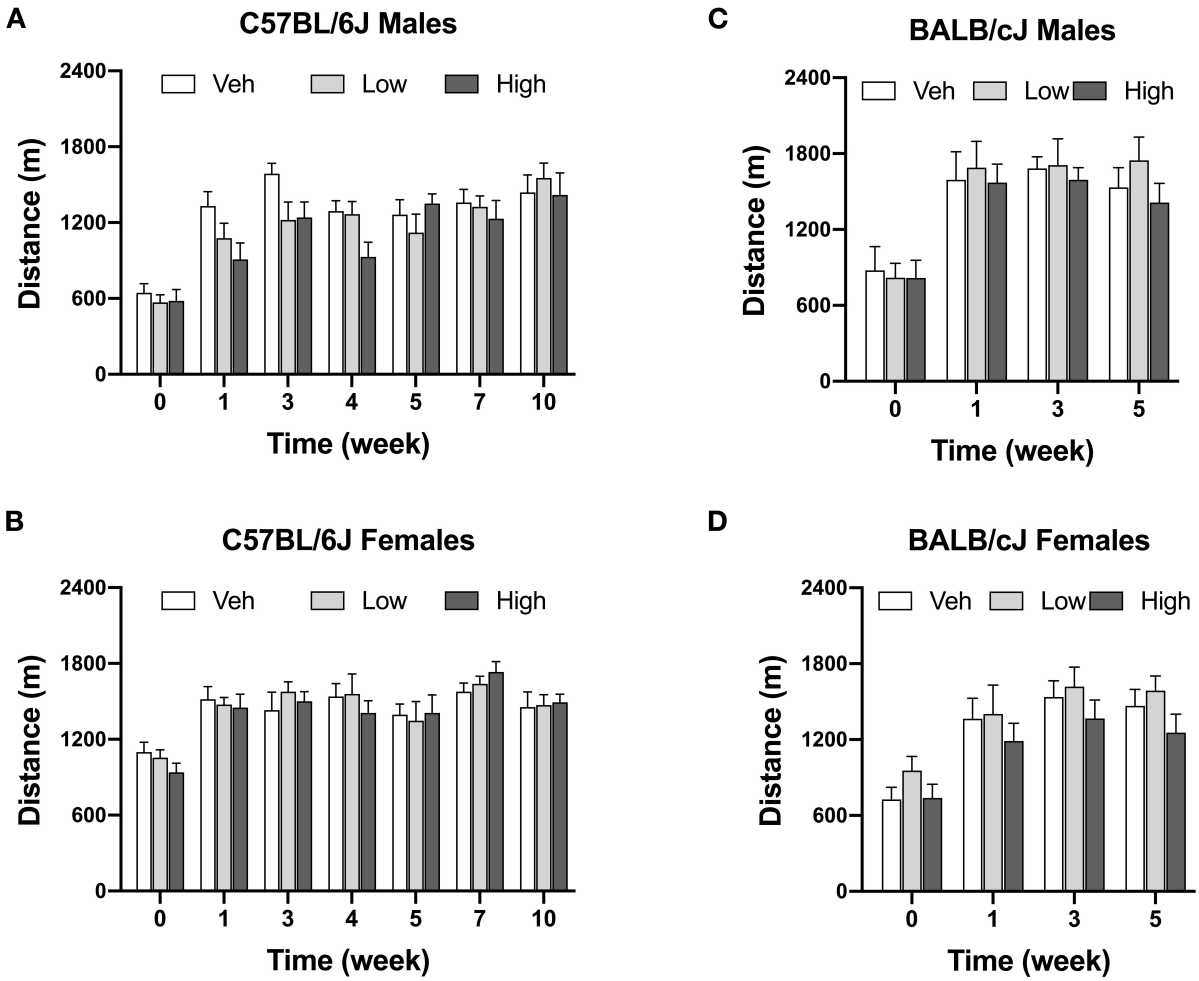
Oxaliplatin has no effect on voluntary wheel running in mice. C57BL/6J males (*n* = 8–10/group) **(A)**, C57BL/6J females (*n* = 6–8/group) **(B)**, BALB/cJ males (*n* = 7–9/group) **(C)**, BALB/cJ females (*n* = 10/group) **(D)**. Values are expressed as mean ± SEM. Results were analyzed using two-way or mixed-analysis model ANOVA (Treatment, Time as RM) for each strain and for each sex followed by Tukey's *post-hoc* test, *p* < 0.05.

Oxaliplatin treatment did not significantly change voluntary wheel running at any of the investigated time points. Male C57BL/6J mice treated with high-dose oxaliplatin were trending toward a decreased in wheel running, however, with no significant interaction between time and dose [*F*_interaction_ (12, 138) = 1.382, *P* = 0.1813] as revealed by two-way ANOVA analysis. Irrespectively of treatment, strain, or sex, animals ran more over time with repeated exposure to the running wheels (Figure 5).

#### Oxaliplatin Reduces Exploration of Light/dark Boxes in Female C57BL/6J Mice

**Figure 6:**
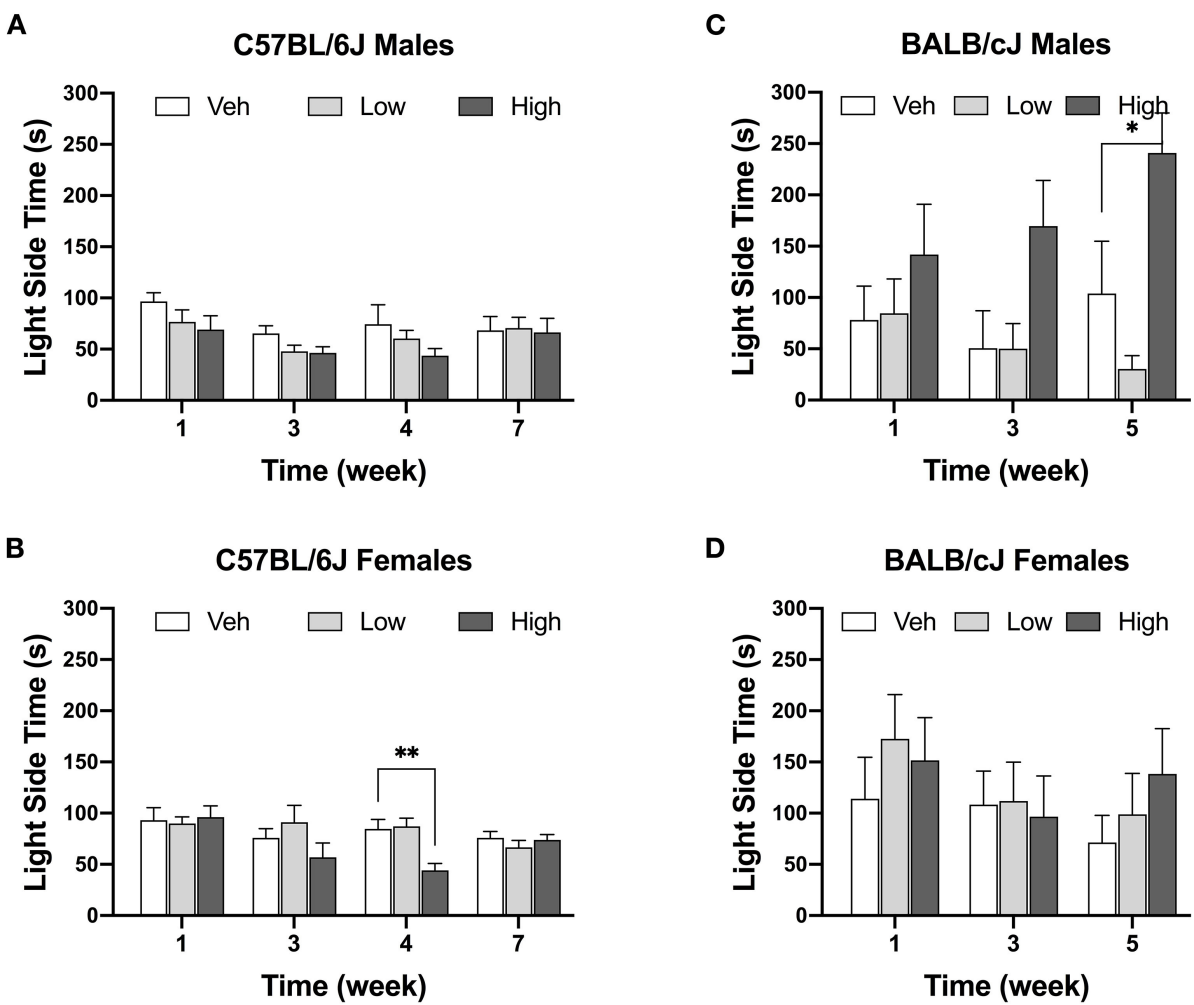
Decrease in time spend in the light area after oxaliplatin exposure. Anxiety-like behavior was assessed in C57BL/6J males (Cohort 1, BL and week 3, *n* = 9/group; Cohort 2, week 4 and week 7, *n* = 8/group) **(A)**, C57BL/6J females (Cohort 1, BL and week 3, *n* = 9/group; Cohort 2, week 4 and week 7, *n* = 8/group) **(B)**, BALB/cJ males (*n* = 7–9/group) **(C)**, BALB/cJ females (*n* = 10/group) **(D)**. Values are expressed as mean ± SEM. Overall effects of oxaliplatin treatment were identified by two-way mixed-analysis model ANOVA (Treatment, Time as RM) for each strain and for each sex ANOVA with *post-hoc* Tukey's multiple comparisons test, *p < 0.05; **p < 0.001.

The high-dose oxaliplatin treatment significantly decreased the time spent in the light compartment of the LDB apparatus only in female C57BL/6J mice at the 4-week time point, as compared to the vehicle controls [*F*_(6, 51)_ = 2.641, *P* = 0.021; veh vs. high dose, *P* = 0.0021]. This effect was transient and by week 7 these animals matched their control group. No effects of oxaliplatin on LDB performance were observed in female BALB/cJ mice (Figure 6D). For BALB/cJ males, there was a trend toward elevated time spent in the light compartment (Figure 6C) with significant effect of dose [*F*_dose_ (2, 21) = 6.479, *P* = 0.0064] an no significant interaction between time and dose [*F*_interaction_ (4, 42) = 1.591, *P* = 0.1944] as revealed by two-way ANOVA analysis. Tukey's multiple comparison test indicated a significant increase in the time spent in the light chamber in male BALB/cJ mice as compared to vehicle controls at week 5 (*P* = 0.025). Testing of BALB/cJ mice was discontinued after week 5 due to equipment malfunction.

#### Oxaliplatin Induces Aversion to 3% Sucrose Solution in the Two-Bottle Choice Test

**Figure 7:**
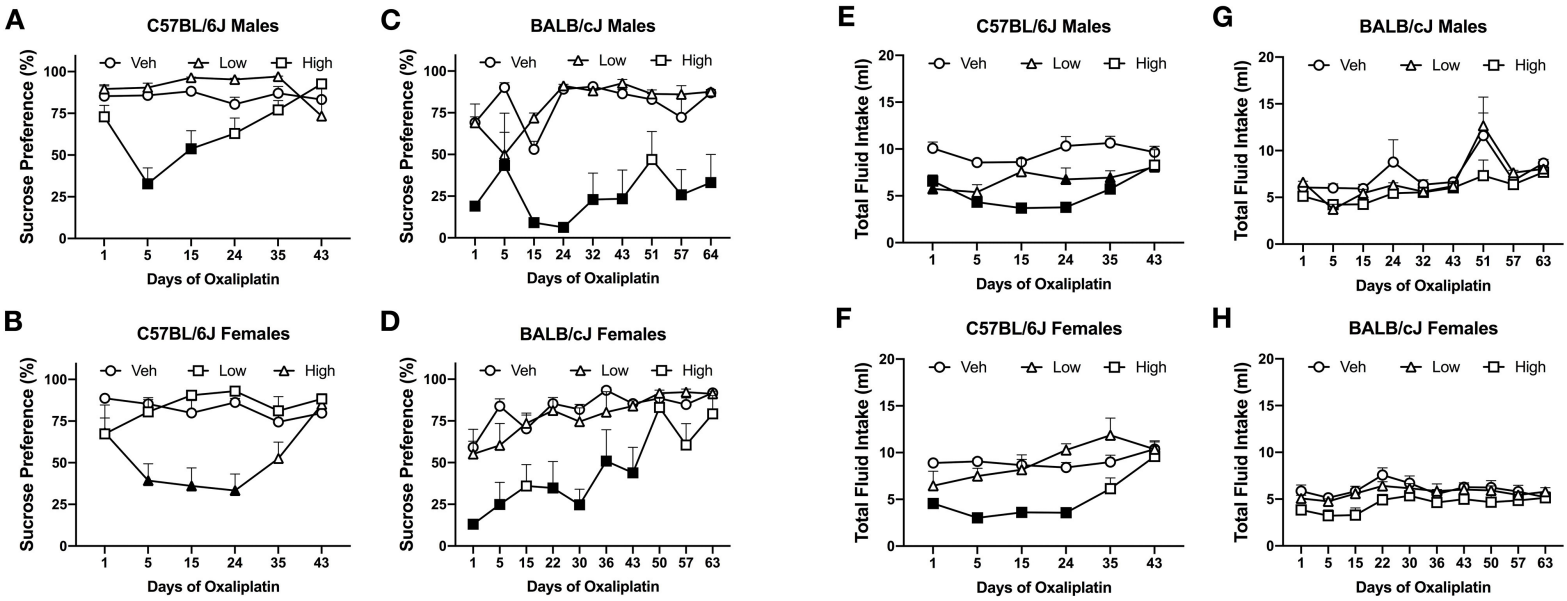
Differential pattern of 3% sucrose solution preference among oxaliplatin-treated animals. Preference was tested in a two-bottle choice test in C57BL/6J males (*n* = 4–10/group) **(A)**, C57BL/6J females (*n* = 4–10/group) **(B)**, BALB/cJ males (*n* = 4–6/group) **(C)**, and BALB/cJ females (*n* = 10/group) **(D)**. The total fluid intake is summarized in C57BL/6J males (*n* = 4–10/group) **(E)**, C57BL/6J females (*n* = 4–10/group) **(F)**, BALB/cJ males (*n* = 4–6/group) **(G)**, and BALB/cJ females (*n* = 10/group) **(H)**. Values are expressed as mean ± SEM. Results were compared using two-way ANOVA (Treatment, Time as RM) for each strain and for each sex followed by Tukey's *post-hoc* test, *p* < 0.05. Filled points indicate times at mechanical hypersensitivity was statistically different from vehicle.

The first 5 injections of high dose oxaliplatin caused a significant drop of sucrose preference in both C57BL/6J and BALB/cJ mice compared to their control groups. C57BL/6J males showed a significant deficit in sucrose preference below on day 5 and 15 [*F*_(10, 105)_ = 4.591, *P* < 0.0001; *P* = 0.0001 and *P* = 0.0037, respectively]. No statistically significant difference was observed in the C57BL/6J males beyond that time point, and the preference recovered to the vehicle group. The mean preference for sucrose in the female C57BL/6J group treated with a high-dose of oxaliplatin was also reduced to 40% after the 5th injection and persisted for two more weeks [*F*_(10, 110)_ = 43.703, *P* = 0.0003, Figure 7B]. Notably, the reduction in sucrose preference by oxaliplatin was more pronounced and lasted much longer in male BALB/cJ than male C57BL/6J mice [*F*_(1, 27)_ = 25.00, *P* < 0.0001, Supplementary Table 1B]. Male BALB/cJ mice showed significantly diminished sucrose preference compared to control mice on day 1, 5, 15, 24, 32, 43, 57, and 64 [*F*_(16, 96)_ = 1.795, *P* = 0.0426] at the high dose of oxaliplatin (Figure 7C). Similarly, BALB/cJ females showed significantly diminished sucrose preference compared to control mice on day 1, 5, 24, 32, and 43 [*F*_(18, 135)_ = 2.116, *P* = 0.0082] upon receiving the high dose regimen of oxaliplatin (Figure 7B). The low-dose of oxaliplatin did not cause significance decrease in sucrose preference in both BALB/cJ male and female mice (Figure 7). High-dose oxaliplatin treated male C57BL/6J mice decreased their fluid intake until 35 days post first administration of the drug while the low-dose treated male C57BL6J mice consumed less fluid on day 1, 24, and 35 [*F*_(10, 105)_ = 3.033, *P* = 0.00021; Figure 7E]. Although, female C57BL/6J mice subjected to the low dose oxaliplatin consumed comparable fluids to the control group, the high dose-treated females reduced drinking on days 1, 5, 15, and 25 [*F*_(10, 110)_ = 3.238, *P* = 0.0011; Figure 7F]. Fluid consumption behavior was not altered by either does of oxaliplatin in BALB/cJ mice (Figures 7G,H).

#### Oxaliplatin Does Not Change Nesting Behavior

**Figure 8:**
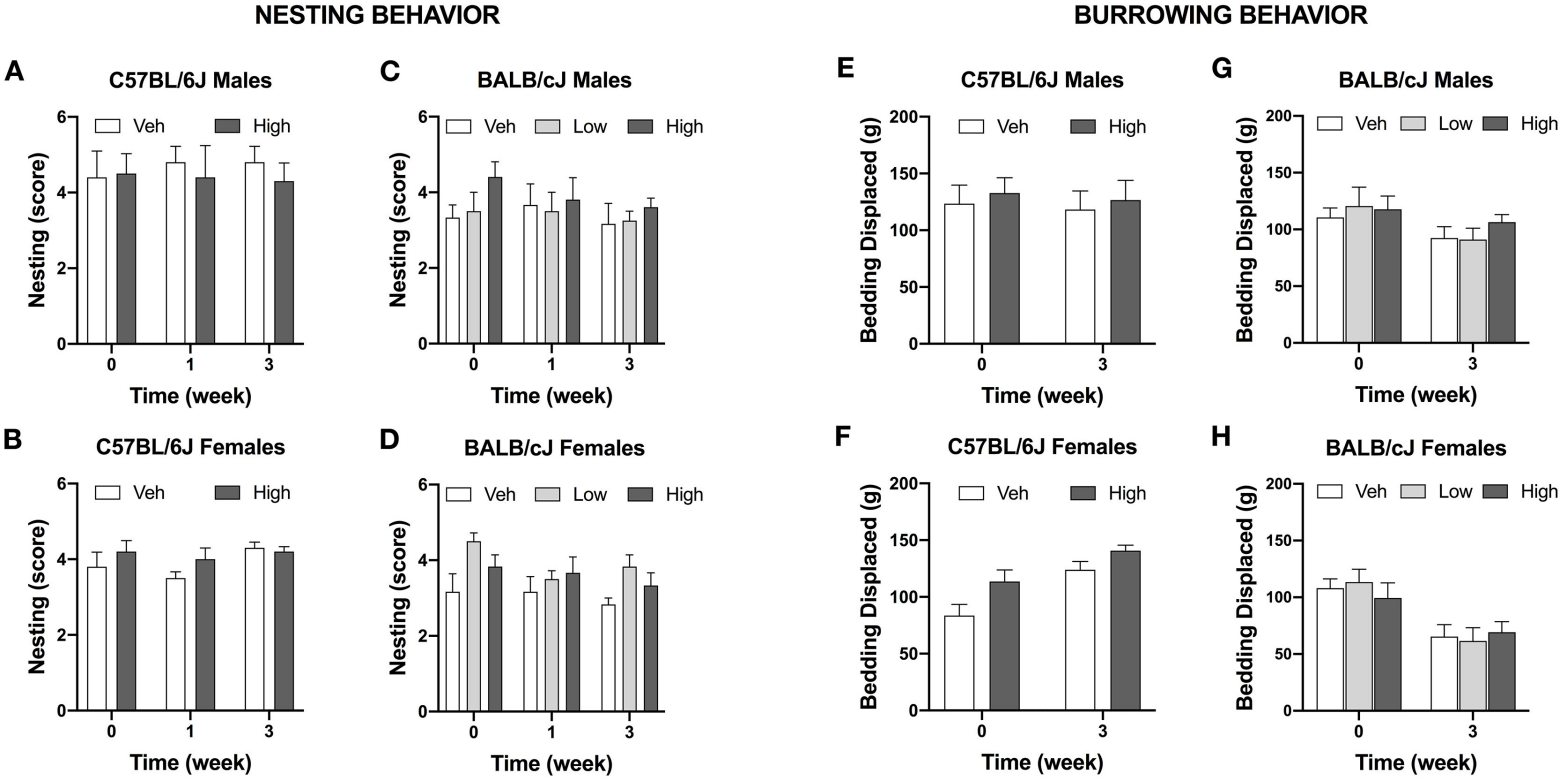
Oxaliplatin has no effect on burrowing and nesting behavior in mice. Nest consolidation after 2 h in C57BL/6J males (*n* = 10/group) **(A)**, C57BL/6J females (*n* = 10/group) **(B)**, BALB/cJ males (*n* = 4–6/group) **(C)**, and BALB/cJ females (*n* = 6/group) **(D)**. Burrowing assessment is depicted for C57BL/6J males (*n* = 10/group) **(E)**, C57BL/6J females (*n* = 10/group) **(F)**, BALB/cJ males (*n* = 4–6/group) **(G)**, BALB/cJ females (*n* = 6/group) **(H)**.

The effect of oxaliplatin on innate behavior was measured in the nest-building tests. The test was administered in the vehicle and high-dose treated C57BL/cJ mice. Neither males nor females C57BL/6J treated with oxaliplatin performed differently than their control groups after completion of each treatment regimen. Similarly, BALB/cJ mice treated with low or high dosage of oxaliplatin were unaffected in their nesting behavior (Figures 8A–D).

#### Oxaliplatin Does Not Change Burrowing Behavior

Oxaliplatin did not alter burrowing activity in C57BL/6J mice when observed after completion of the first and second treatment regimens [*F*_(1, 18)_ = 0.00171, *P* = 0.9674, males; *F*_(1, 18)_ = 0.7891, *P* = 0.0.3861, females; Figures 8E,F]. Comparably, oxaliplatin treatment had no effect on bedding displacement in either sex of BALB/cJ mice at week 3 [*F*_(2, 11)_ = 0.4666, *P* = 0.6390, males; *F*_(2, 27)_ = 0.9337, *P* = 0.4054, females; Figures 8G,H].

#### Oxaliplatin Has Sex- and Strain-Dependent Effects on Caudal Nerve Conductance

**Figure 9:**
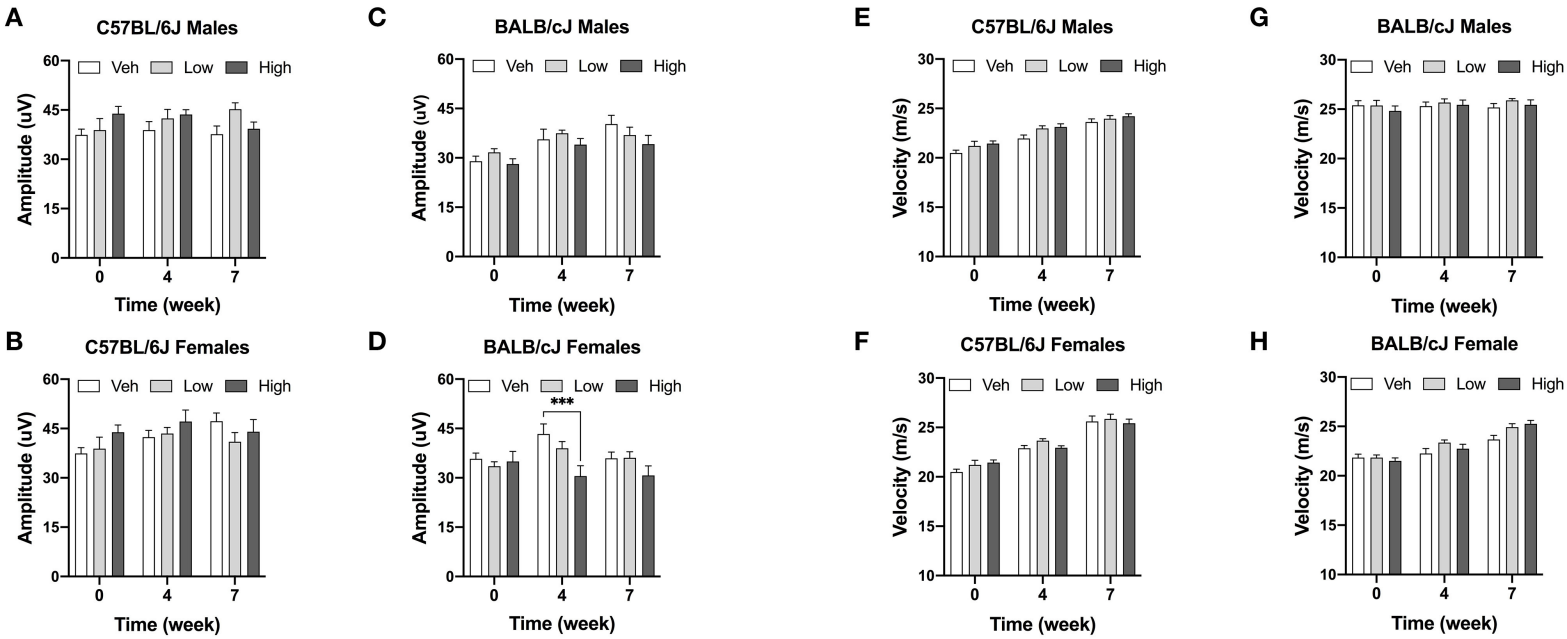
Electrophysiological analysis of the caudal sensory nerves. One-week post completion of oxaliplatin or vehicle treatment, sensory nerve conduction amplitude **(A–D)** and velocity **(E–H)** were measured in C57BL/6J males and females (*n* = 8/group) and BALB/cJ males and females (*n* = 7–10/group). Values are expressed as mean ± SEM. Overall effect of oxaliplatin treatment per sex of each strain was identified using two-way ANOVA and *post-hoc* Tuckey's test (***p < 0.001).

Caudal NCV and SNAP of male and female C57BL/6 and BALB/cJ were measured to determine the functional effect of oxaliplatin on peripheral sensory nerves at baseline (pretreatment and 4 and 7 weeks post-treatment). First, we determined sex and strain differences in nerve conduction amplitude and velocity at baseline. The SNAP and NCV of male and female C57BL/6J mice was statistically different, while male BALB/cJ mice had a lower SNAP and higher NCV than female BALB/cJ mice [*F*_(3, 96)_ = 13.25, *P* < 0.0001; *P* = 0.0233, *P* < 0.0001]. Moreover, strain differences in the nerve conduction amplitude differed between both sexes (Supplementary Figure 1B). Male and female C57BL/6J mice had a significantly higher amplitudes than male and female BALB/cJ mice, respectively (Supplementary Figure 1A: *P* < 0.0001 male C57BL/6J vs. BALB/cJ; Supplementary Figure 1B: *P* = 0.0233 female C57BL/6J vs. female BALB/cJ), and the baseline NCV of male C57BL/6J was lower than male BALB/cJ mice (*P* < 0.0001) One-way ANOVA revealed a significant impact of high-dose oxaliplatin on SNAP in BALB/cJ females compared to vehicle group at week 4 [*F*_(4, 54)_ = 2.888, *P* = 0.001, Figure 9D]. No significant changes were observed in nerve conduction velocities post oxaliplatin treatment (Figures 9E–H).

#### Oxaliplatin Treatment Reduces Intraepidermal Nerve Fiber Density in the Ventral Surface of the Hind Paw

**Figure 10:**
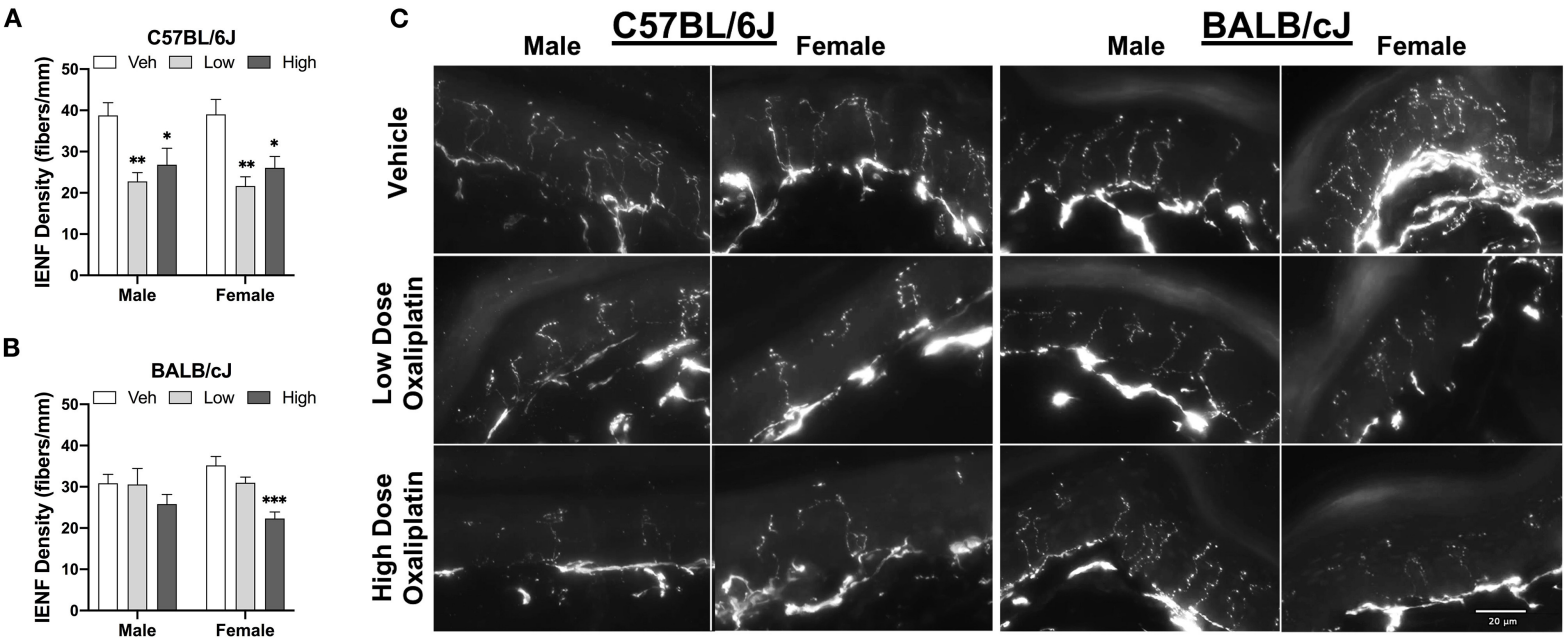
Reduction of intraepidermal nerve fiber density in the hind paw at week 11. **(A)** shows C57BL/6J males and females (*n* = 6/group), while changes to IENF density in BALB/cJ males and females (*n* = 6/group) are shown in **(B)**. Representative fluorescence microscopic images of IENF taken at 40X **(C)**. Values are expressed as mean ± SEM. Overall effects of oxaliplatin treatment per sex of each strain were identified using one-way ANOVA Treatment) for each strain and for each sex, and post-hoc Dunnet's test (*p < 0.05; **p < 0.001; ***p < 0.0001).

The density of IENFs was evaluation in hind paw samples after completion of behavioral testing at week 11. Both doses of oxaliplatin significantly reduced IENF density in male C57BL/6J mice, as compared to vehicle control [One-way ANOVA: *F*_(2, 16)_ = 7.311, *P* = 0.0035, low vs. veh *P* = 0.0035, high vs. veh *P* = 0.0298]. Comparably, C57BL/6J females presented a similar pattern of the nerve fiber loss [One-way ANOVA: *F*_(2, 15)_ = 9.437, *P* = 0.0022, low vs. vehicle *P* = 0.0015, high vs. vehicle *P* = 0.0132; Figure 10A]. A dose-related decrease of IENF density was observed in female BABL/cJ mice where only the high-dose treated mice demonstrated reductions in the density of IENFs when compared to vehicle-treated group [One-way ANOVA: *F*_(2, 15)_ = 14.3, *P* = 0.0003; Figure 10B]. However, BALB/cJ males treated with the anti-tumor drug had similar density of IENF to the control group. Representative foot pad sections from stained for PGP9.5 protein show the variation in IENFs density following oxaliplatin treatment (Figure 10C).

## Discussion

Oxaliplatin is a platinum-based chemotherapeutic agent widely used for colorectal cancer therapy and other gastrointestinal tumors, but its use is associated with various adverse effects, including OIPN (10, 36). Clinically, OIPN appears in two distinct forms: an acute and transient neuropathy that develops in almost 90% of patients within hours of infusion that can last up to 7 days, and in 40–80% of patients develop a chronic dose-limiting cumulative sensory neuropathy (5). OIPN is also associated with functional impairment and affective disorders (depression and anxiety) induced by the drug, reducing the quality of life for many cancer patients (37–39). Pre-clinical models play a pivotal role in understanding the pathophysiology of OIPN and in testing potential therapies. Unfortunately, this translational effort has not been very successful, and no effective preventive treatment exists. In addition, pharmacological management of OIPN related pain remains very challenging. Possible reasons for this could include limitations and inconsistencies in the experimental design of animal studies, such as the use of single dosing regimen, different testing times post treatment, the lack of testing in both sexes, and the genetic background of animals. In addition, the use of outcome measures and behavioral endpoints other than sensory gain, that are translatable to the human pain-related experiences is not common (40).

In consideration of the aforementioned factors, this study examined alterations in the nociceptive, behavioral, and morphological aspects of OIPN in both sexes of C57BL/6J and BALB/cJ mice. The impact of oxaliplatin on affective-like behaviors was measured at multiple time points via battery of testing, which included light/dark boxes, sucrose preference, wheel running, nesting, and burrowing. Overall, our results demonstrate that C57BL/6J mice seem more sensitive to oxaliplatin-induced behavioral changes compared to BALB/cJ mice, with the exception of change in preference for sucrose solution. In addition, oxaliplatin had sex-, dose-, and strain-dependent effects on caudal nerve conduction and IENF degeneration. Furthermore, none of the low-dose regimen treated mice showed changes in affective-like behaviors.

Sensory nociceptive changes in rodents are routinely assessed via cold and mechanical hypersensitivity. Administration of 5 injections of the low oxaliplatin dose triggered mechanical hypersensitivity at week 1 in male and female BALB/cJ mice. In contrast, male C57BL/6J but not female C57BL/6J mice developed mechanical hypersensitivity at week 1. Regardless of sex or strain, all mice exhibited mechanical hypersensitivity after completion of the high-dose regimen of oxaliplatin. Treatment with the low and high doses of oxaliplatin produced significant mechanical hypersensitivity that persisted throughout the observation period of 10 weeks. In the same OIPN model, Ta et al. (11) reported mechanical hypersensitivity lasted until week 6 of the experimental timeline in male C57BL/6J mice treated with the high dose of oxaliplatin. However, in Ta's et al. measurements were carried out with electronic filaments, while we used manual filaments. In regard to cold and mechanical hypersensitivity, the literature on oxaliplatin-induced peripheral neuropathy shows heterogeneity in terms of experimental set up, dose, and route of administration (41, 42).

Cold-sensitive sensory symptoms is a main clinical feature of OIPN (43). In line with clinical and animal data, our results show time- and dose-dependent cold hypersensitivity in both sexes of C57BL/6J mice. Male C57BL/6J mice showed an increase in response to acetone after the 1st week of high-dose oxaliplatin treatment that persisted for at least 1 week after cessation of the treatment regimen. Similarly, Meade et al. (44) demonstrated that oxaliplatin causes cold hypersensitivity in male and female C57BL/6J mice. Administration of cumulative 3 mg/kg of oxaliplatin spread over 15 days resulted in cold hypersensitivity that lasted for 1 week after last injection. Interesting, a single bolus of 3 mg/kg i.p. induced cold hypersensitivity in the acetone test that persisted for 3 weeks in male C57BL/6 mice (45). One research group demonstrated that BALB/c mice treated with intravenous oxaliplatin may have greater cold hypersensitivity than C57BL/6 mice in the cold plate test (20). However, we were not able to assess cold hypersensitivity with the acetone test in female and male BALB/cJ mice due to excessive hyperactivity on the mesh testing apparatus.

Degeneration of intraepidermal nerve fibers has been implicated in neuropathic pain in preclinical and clinical studies (46–48). IENFs are free nerve endings originate from unmyelinated and thinly myelinated nociceptors within the dermis. They play and important role in transmission of peripheral pain (49). Various classes of chemotherapeutics have been shown to cause structural changes and degradation of unmyelinated nerve fibers, suggesting a common etiology of CIPN (50, 51). With respect to IENF changes, we observed that C57BL/6J mice showed differential degeneration of IENF compared to BALB/cJ mice as both the low and high oxaliplatin doses caused a significant reduction of IENF density in C57BL/6J mice 8 weeks after cessation of treatment. BALB/cJ mice showed sex-dependent decrease of the fibers' density: only the female BALB/cJ mice treated with the high dose of oxaliplatin had a significant degeneration of nerve fibers. In contrast to our findings, the work of Marmiroli et al. (20) shows that BALB/c mice are more susceptible to the effects of intravenous treatment with 28 mg/kg oxaliplatin on IENF than C57BL/6, 1 week post treatment completion (20). The discrepancy between these results could be explained by difference in timing of tissue collection, route of administration, and C57BL/6 substrain differences that could affect these results (52). Indeed, we and others recently reported that C57BL/6J and C57BL/6N substrains differ in their responses in acute and tonic pain models (53–55).

Preclinical research on chemotherapy-induced peripheral neuropathy points to involvement of pro-inflammatory cytokines in modulation of peripheral nerve damage. Upon treatment with chemotherapy, tumor necrosis factor alpha (TNFα), IL-1 β, and IL-6, and interferons alpha and gamma are all elevated (56–59). These cytokines contribute greatly to both pain and damage to neuron and supporting cells. Taken together, this suggest that neuropathies affect not just the peripheral nerves, but also the homeostasis of the skin. The innate immune response in C57BL/6 and BALB/c has been shown to differ. Upon with macrophage-activating lipopeptide-2 or lipopolysaccharide, macrophages derived from female C57BL/6 mice produced higher levels of TNF-alpha and interleukin than macrophages female BALB/c mice macrophages (60). Additionally, proinflammatory cytokine production is sexually dimorphic and variable depending on the genetic background of mice (61). Underlying differences in immunological responses between the two strains might explain this phenomenon. Further, it is possible that sub-strain genetic variations might play an important role in the degree of degradation of IENF due to oxaliplatin treatment. Presence of mechanical hypersensitivity, despite loss of IENF, might be mediated by hyperactive and/or sensitized nociceptive fibers sensitized resulting from the release of chemical mediators of inflammation (62).

Oxaliplatin highly impacts peripheral sensory neurons (63). To measure alterations in peripheral nerve function and potential degradation of the myelinated fibers, the amplitude and velocity of the sensory caudal nerve conductions were measured. Our results indicate that the SNAP was significantly affected by the high dose of oxaliplatin in female BALB/cJ mice only. Similarly, 28 mg/kg intravenous oxaliplatin reduced the action potential amplitude in tails of female BALB/c mice (42). Platinum compounds, such as oxaliplatin, irreversibly bind to DNA thus inducing damage and apoptosis of primary sensory neurons (64). While sensory nerve conduction examines large-fiber neuropathy, skin innervation is a parameter of small-fiber sensory neuropath (65). CIPN patients diagnosed with loss of IENF can have normal nerve conduction results (66, 67). Correspondingly, in our study, C57BL/6J mice showed reduced mechanical hypersensitivity and IENF density but no changes in the caudal sensory nerve amplitude. The results of a quantitative analysis by Marmiroli et al. (20) reports significant reduction of DRG neuronal structures upon oxaliplatin treatment. However, DRG from oxaliplatin-treated C57BL6 mice did not different from their naïve controls. This further confirms that oxaliplatin impairment of neurophysiological morphology and functions is distinctive among different strains of mice.

Body weight can be an indication of rodent welfare. Male C57BL/6J mice treated with high-dose regimen of oxaliplatin lost more weight and took longer to return to baseline body weights than female C57BL/6J or male BALB/cJ mice (Supplementary Tables 1A,B). The high dose of oxaliplatin caused a significant decrease in BALB/cJ females 1 week post last administration of the antineoplastic agent. Reduction of body weights or slower body gain due to platinum-based chemotherapeutics, such as oxaliplatin, was noted by others in male rats and C57BL/6J mice and BALB/c mice (68–70). It has been shown that male C57BL/6J mice treated with the same treatment regimen of oxaliplatin experienced skeletal myopathy (71). Both male and female C57BL/6J mice decreased their spontaneous locomotor activity. A similar finding was observed in male C57BL/6J mice with reduced locomotor activity (11). The changes in body weight and locomotor disturbances indicate that BALB/cJ mice are less sensitive to oxaliplatin-induced toxicity.

Pain is a complex phenomenon that has both sensory and emotional components (72). Additionally, reflexive reactions to nociceptive stimuli do not necessarily indicate the experience of spontaneous pain in humans (22). According to epidemiological studies, the prevalence of depression may be as high as 85% in patients suffering from chronic pain (73, 74), and around 30% for patients suffering from neuropathic pain (75). Individuals diagnosed with depression report increased frequency and severity of acute pain. Furthermore, the magnitude and duration of acute pain has indicated higher level of anxiety and depression in people (76). Pre-clinical models of nerve injury induced by mechanical, chemotherapeutic, or inflammatory insults saw changes in affective behavior suggestive of a depression-like state (23, 77, 78). Correspondingly, it has been reported that male C57BL/6J mice treated single i.p., dose of 6 mg/kg oxaliplatin showed an increased immobility time in forced swim test, suggesting that oxaliplatin can induce depressive-like behavior in rodents (79).

In our study, the low dose of oxaliplatin did not affect the behavioral assays measured at the tested time points in mice in both mouse strains. The high-dose of oxaliplatin had some impact on female C57BL/6J. We show that female C57BL/6J treated with high-dose of oxaliplatin spent significantly more time in the dark side, indicating an increase in anxiety-like behavior. This is first study to report testing anxiety-like behavior via light/dark box in mice treated with oxaliplatin. Anxiety-like behavior was measured and observed in a paclitaxel-induced peripheral neuropathy model. Repeated administration of the chemotherapeutic drug in C57BL/6J males caused a long-lasting mechanical hypersensitivity and anxiety-like phenotype, as observed 9 weeks post treatment (23). Two studies examining the impact of oxaliplatin on neurotoxicity in male Swiss mice and male Sprague-Dawley rats demonstrated reduction of time spent in the open arms and the percent of open arm entries in an elevated plus-maze assay, indicative of anxiety-like state (80, 81). Moreover, male C56BL/6J mice treated with oxaliplatin took a longer time to reach the center in a novelty suppressed feeding test; however, no difference in elevated plus maze were observed (82, 83). In a spared nerve injury model, female mice were identified as more susceptible to anxiety-like behavior (84). Conversely, oxaliplatin did not alter wheel running, nesting nor burrowing behaviors. These assays have been shown to reflect motivation and innate behaviors that are used to assess general well-being of rodents. While BALB/c and C57BL/6 mice are the most commonly used strain in anxiety and biomedical studies (24), the literature is inconsistent on which stain is more anxious (85). Male BALB/cJ mice exhibited elevated time spent in the light compartment. This could have been attributed to freezing behavior, a sign of anxiety-like behavior. The general activity of these animals was not affected, as indicated by locomotor activity test results, however between-strain analysis identified significant difference between female C57BL/6J and BALB/cJ mice treated with high dose of oxaliplatin (Supplementary Table 1B). Overall, detection of behavioral depression of mice treated with oxaliplatin might be dependent on the assay. Characteristic phenotypical differences exist across mouse strain and they should be considered when selecting strain and sex of the rodents.

A large and long-term deficit was observed after oxaliplatin administration in the sucrose preference test. Rodents show a natural preference for the sweet taste (86), and a deficit in sucrose preference has been shown to be sensitive to different animal models of depression (87, 88). We observed a decreased preference for a 3% sucrose solution in both C57BL/6J and BALB/cJ mice treated with the high-dose oxaliplatin regimen. Oxaliplatin treatment produced a longer-lasting reduction of sucrose preference in BALB/cJ males than C57BL/6J males, even though both had significant mechanical hypersensitivity for the duration of the study (Supplementary Table 1B). The decrease in sucrose seen in C57BL/6J males and female mice at the higher dose regimen of oxaliplatin may be the consequence of a general decrease in drinking behavior. It should be noted that a significant decrease in body weight was noted in these oxaliplatin-treated mice. Future studies assessing quinine and saccharine preference will be useful to understand if oxaliplatin's effects on sucrose preference in C57BL/6J mice are not due to sensory loss. Total fluid intake was not altered in BALB/cJ mice undergoing the high-dose oxaliplatin regimen. It is possible that the sucrose preference deficit seen reflects taste alteration by oxaliplatin in mice. In the clinic, about 12% of patients receiving oxaliplatin report alteration in taste (89). However, in a pre-clinical paclitaxel-induced CIPN model, Toma et al. (23) reported a transient reduction sucrose preference in C57BL/6J males accompanied by time-dependent changes in other affect-like behaviors. The aversion to sucrose preference was proposed to be mediated by kappa opioid receptor (KOR) signaling dysregulation, as the selective KOR antagonist norbinaltorphimine was able to reversible sucrose preference deficit (90). Another explanation for changes in sucrose preference is gustatory alteration induced by oxaliplatin. However, in our recent study, C57BL/6J and BALB/cJ mice subjected to the same high-dose oxaliplatin schedule consumed similarly number of sucrose pellets as the vehicle control group (44).

In this study, we looked at the differences in the development of oxaliplatin-induced neuropathy in male and female mice. The specific alterations in affect-like behaviors, IENF density, and nerve conduction amplitude show different profiles of C57BL/6J and BALB/cJ mice. However, these changes are not uniform across strains indicating strain differences as well. A three-way ANOVA with repeated measures was performed to investigate the interaction between sex, treatment, and time (Supplementary Table 1A). A significant difference in percent body weight of female and male was observed in C57BL/6J mice treated with the high dose of oxaliplatin and their control vehicle. We were not able to determine whether the cause of the body weight loss in C57BL/6J mice was caused by a decrease in fluid intake or reduction of lean and fat tissue mass as suggested Sorensen et al. (71). Regarding sex differences between male and female BALB/cJ mice, two behavioral assays showed trending statistical tendencies. BALB/cJ males tended to spent more time in the light compartment than the female BALB/cJ mice [*F*_(1, 95)_ = 2.774, *P* = 0.0991; Supplementary Table 1A]. The second trending difference between male and female BALB/cJ mice treated with the high dose of oxaliplatin was alteration in in IENF density [*F*_(1, 20)_ = 3.549, *P* = 0.0742]. While BALB/cJ females exhibited a significant reduction of IENF, male BALB/cJ mice had comparable IEND to their control group. One possible explanation of these sex differences is the role of hormonal mechanisms of OIPN. These differences can also be due to genetic and neuroimmune mechanisms of OIPN processing in male and female mice such as the release of pronociceptive substances substance P and calcitonin gene-related peptide, as well as exposure to pro-inflammatory cytokines released by infiltrating and resident immune cells (63).

This study has some limitations. While the primary aim of this study was to compare our results to literature on rodent studies using the same route of administration, oxaliplatin delivery method via intraperitoneal injections poses some translational limitations. The absorption, distribution, metabolism, or excretion of chemotherapeutic agents have been shown to influence the development of CIPN in human patients (91, 92). Moreover, a pharmacokinetics study in rats showed significant differences in the AUC for peritoneal fluid and plasma drug concentrations after 90 min post treatment with 5 mg/kg of oxaliplatin (93). A future study assessing pharmacokinetics of oxaliplatin via intraperitoneal and intravenous routes of administration in C57BL/6J and BALB/cJ would be beneficial and could explain some of the observed differences. Additionally, it is plausible that the early time point behavioral alterations are associated with loss of IENF, hence, it would be useful to assess IENF density at week 3 or 5.

## Conclusions

We demonstrated that affective-like behaviors in male and female C57BL/6J and BALB/cJ mice were differentially ad dose-dependently affected by oxaliplatin. We demonstrate that the low and high treatments of oxaliplatin induced a long-lasting mechanical hypersensitivity that did not largely correlate with deficit in affect-like behaviors.

## Data Availability Statement

The original contributions presented in the study are included in the article/Supplementary Material, further inquiries can be directed to the corresponding author/s.

## Ethics Statement

The animal study was reviewed and approved by Institutional Animal Care and Use Committee of Virginia Commonwealth University.

## Author Contributions

UW: conceptualization, data curation, formal analysis, investigation, methodology, writing—original draft, and review & editing. WT and JM: data curation, formal analysis, methodology, and writing—review & editing. AP and DT: data curation, investigation, and writing—review & editing. MC, JB, and CB: formal analysis and writing—review & editing. MD: conceptualization, formal analysis, funding acquisition, methodology, project administration, resources, supervision, and writing—review & editing. All authors contributed to the article and approved the submitted version.

## Conflict of Interest

The authors declare that the research was conducted in the absence of any commercial or financial relationships that could be construed as a potential conflict of interest.
