# Supplementary material for: Impact of Dose, Sex, and Strain on Oxaliplatin-Induced Peripheral Neuropathy in Mice
Source: Front Pain Res (Lausanne). 2021 Jul 22;2:683168. doi: 10.3389/fpain.2021.683168 (PMC8915759; doi:10.3389/fpain.2021.683168)
Supplement: Supplementary file 1 [file Data_Sheet_1.DOCX]

**Supplementary Figure 1.** **Baseline Electrophysiological analysis of the caudal sensory nerves**. Sensory nerve conduction amplitude(A) and velocity (B) were measured in C57BL/6J males and females (n = 8/group), and BALB/cJ males and females (n = 7-10/group) respectively. Values are expressed as mean ± SEM. Overall effects of oxaliplatin treatment per sex of each strain were identified using one-way ANOVA (Treatment) for each strain and for each sex, and post-hoc Tuckey's test (* p < 0.05, *** p < 0.0001).

**Supplementary** **Table 1**. A three-way ANOVA with repeated measures was performed to investigate the interaction between a) sex x treatment x time; and b) strain x treatment x time.
