# Supplementary figures and images for: Impact of Dose, Sex, and Strain on Oxaliplatin-Induced Peripheral Neuropathy in Mice
Source: Front Pain Res (Lausanne). 2021 Jul 22;2:683168. doi: 10.3389/fpain.2021.683168 (PMC8915759; doi:10.3389/fpain.2021.683168)

**A**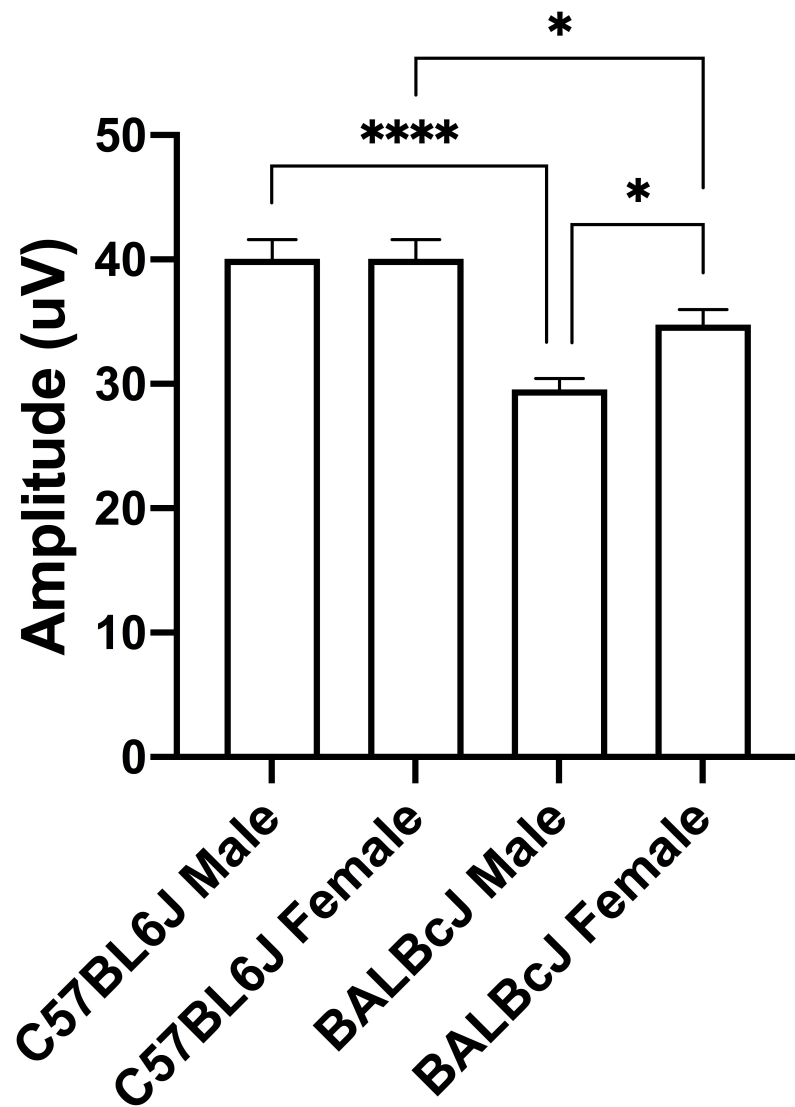**B**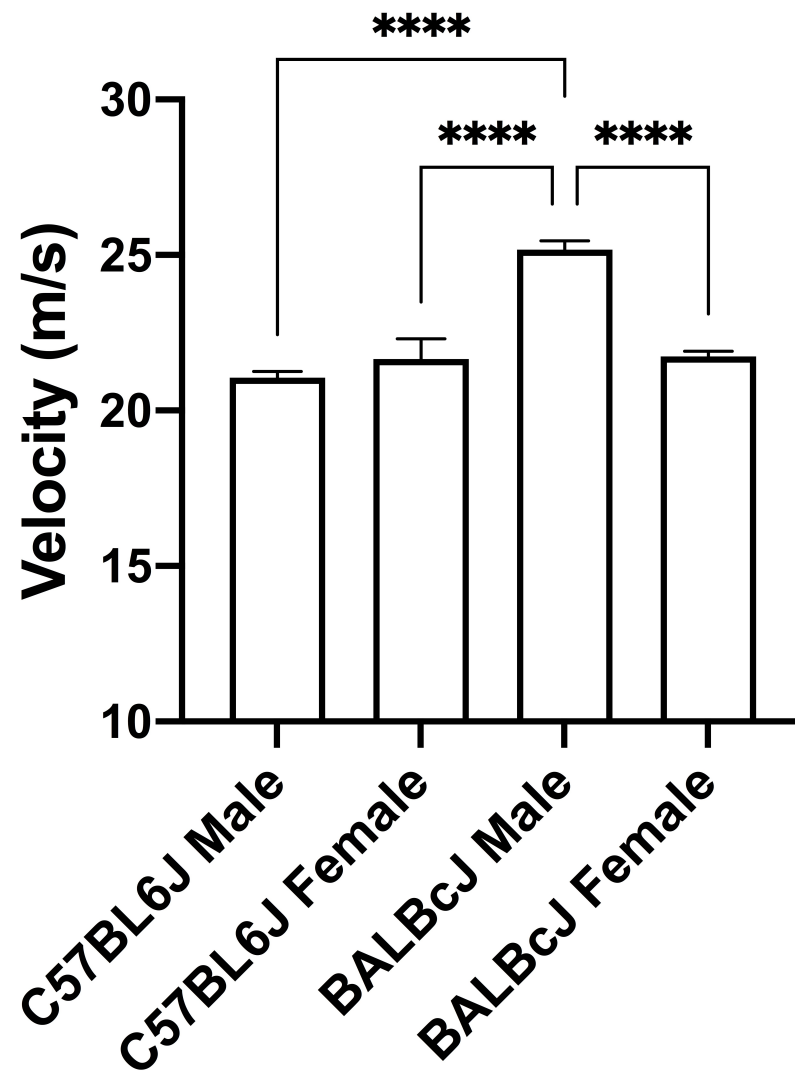

Supplement: Supplementary file 2 [file Image_1.pdf]
