# Supplementary material for: Impact of Dose, Sex, and Strain on Oxaliplatin-Induced Peripheral Neuropathy in Mice
Source: Front Pain Res (Lausanne). 2021 Jul 22;2:683168. doi: 10.3389/fpain.2021.683168 (PMC8915759; doi:10.3389/fpain.2021.683168)
Supplement: Supplementary file 3 [file Table_1.pdf]

A

Measurement	Males vs. Females	Treatment	Sex x Treatment
Percent Body Weight Change	C57BL/6J	Veh vs Low Dose	F (1, 52) = 0.03851; P=0.8452
		Veh vs High Dose	F (1,52)= 6.360; P=**0.0148**
	BALB/cJ	Veh vs Low Dose	F (1, 31) = 1.400; P=0.2458
		Veh vs High Dose	F (1, 33) = 0.04765; P=0.8286
Mechanical Hypersensitivity	C57BL/6J	Veh vs Low Dose	F (1, 53) = 1.122; P=0.2943
		Veh vs High Dose	F (1, 55) = 2.472; P=0.1216
	BALB/cJ	Veh vs Low Dose	F (1, 31) = 0.02937; P=0.8650
		Veh vs High Dose	F (1, 31) = 0.03572; P=0.8513
Cold Hypersensitivity	C57BL/6J	Veh vs Low Dose	F (1, 28) = 0.2260; P=0.6382
		Veh vs High Dose	F (1, 28) = 0.2858; P=0.5972
Locomotor Activity	C57BL/6J	Veh vs Low Dose	F (1, 224) = 0.5713; P=0.4505
		Veh vs High Dose	F (1, 226) = 1.116; P=0.2919
Light/Dark Box	C57BL/6J	Veh vs Low Dose	F (1, 116) = 0.2847; P=0.5947
		Veh vs High Dose	F (1, 116) = 0.5115; P=0.4759
	BALB/cJ	Veh vs Low Dose	F (1, 29) = 0.7639; P=0.3893
		Veh vs High Dose	F (1, 95) = 2.774; P=0.0991
Sucrose Preference	C57BL/6J	Veh vs Low Dose	F (1, 25) = 0.4840; P=0.4930
		Veh vs High Dose	F (1, 36) = 1.721; P=0.1979
	BALB/cJ	Veh vs Low Dose	F (1, 18) = 0.2419; P=0.6288
		Veh vs High Dose	F (1, 19) = 2.439; P=0.1349
Total Fluid Intake	C57BL/6J	Veh vs Low Dose	F (1, 25) = 10.56; P=**0.0033**
		Veh vs High Dose	F (1, 36) = 0.1929; P=0.6631
	BALB/cJ	Veh vs Low Dose	F (1, 19) = 0.03389; 0.8559
		Veh vs High Dose	F (1, 19) = 0.005325; 0.9426
Nerve Conduction Amplitude	BALB/cJ	Veh vs Low Dose	F (1, 30) = 0.6407; P=0.4298
		Veh vs High Dose	F (1, 31) = 0.7612; P=0.3897
IENF Density	C57BL/6J	Veh vs Low Dose	F (1, 21) = 0.06181; P=0.8061
		Veh vs High Dose	F (1, 20) = 0.02172; P=0.8843
	BALB/cJ	Veh vs Low Dose	F (1, 20) = 0.5786; P=0.4557
		Veh vs High Dose	F (1, 20) = 3.549; P=0.0742

B

Measurement	C57BL/6J vs. BALB/cJ	Treatment	Strain x Treatment
Percent Body Weight Change	Males	Veh vs Low Dose	F (1, 39) = 1.929; P=0.1728
		Veh vs High Dose	F (1, 41) = 21.39; P<**0.0001**
	Females	Veh vs Low Dose	F (1, 44) = 0.5143; P=0.4771
		Veh vs High Dose	F (1, 44) = 0.3482; P=0.5582
Mechanical Hypersensitivity	Males	Veh vs Low Dose	F (1, 46) = 0.008642; P=0.9263
		Veh vs High Dose	F (1, 49) = 0.2708; P=0.6051
	Females	Veh vs Low Dose	F (1, 38) = 0.5723; P=0.4540
		Veh vs High Dose	F (1, 37) = 0.4533; P=0.5050
Locomotor Activity	Males	Veh vs Low Dose	F (1, 128) = 3.475; P=0.0646
		Veh vs High Dose	F (1, 138) = 1.592; P=0.2092
	Females	Veh vs Low Dose	F (1, 104) = 0.2369; P=0.6275
		Veh vs High Dose	F (1, 104) = 7.881; P=**0.0060**
Light/Dark Box	Males	Veh vs Low Dose	F (1, 23) = 0.1954; P=0.6626
		Veh vs High Dose	F (1, 25) = 3.199; P=0.0858
	Females	Veh vs Low Dose	F (1, 51) = 0.2634; P=0.6100
		Veh vs High Dose	F (1, 27) = 0.09829; P=0.7563
Sucrose Preference	Males	Veh vs Low Dose	F (1, 20) = 2.029; P=0.1697
		Veh vs High Dose	F (1, 27) = 25.00; P<**0.0001**
	Females	Veh vs Low Dose	F (1, 23) = 0.8560; P=0.3645
		Veh vs High Dose	F (1, 28) = 2.759; P=0.1079
Total Fluid Intake	Males	Veh vs Low Dose	F (1, 22) = 6.307; P=**0.0199**
		Veh vs High Dose	F (1, 161) = 21.87; P<**0.0001**
	Females	Veh vs Low Dose	F (1, 23) = 0.4342; P=0.5165
		Veh vs High Dose	F (1, 28) = 6.314; P=**0.0180**
Nerve Conduction Amplitude	Females	Veh vs Low Dose	F (1, 32) = 0.1114; P=0.7408
		Veh vs High Dose	F (1, 32) = 5.241; P=**0.0288**
IENF Density	Males	Veh vs Low Dose	F (1, 21) = 7.535; P=**0.0121**
		Veh vs High Dose	F (1, 20) = 1.342; P=0.2603
	Females	Veh vs Low Dose	F (1, 20) = 6.995; P=**0.0155**
		Veh vs High Dose	F (1, 20) = 0.0004404; P=0.9835
